# Hemoglobin Glycation Index: A Novel Risk Factor for Incident Chronic Kidney Disease in an Apparently Healthy Population

**DOI:** 10.1210/clinem/dgad638

**Published:** 2023-11-01

**Authors:** Yasuto Nakasone, Takahiro Miyakoshi, Takahiro Sakuma, Shigeru Toda, Yosuke Yamada, Tomomasa Oguchi, Kazuko Hirabayashi, Hideo Koike, Koh Yamashita, Toru Aizawa

**Affiliations:** Diabetes Center, Aizawa Hospital, Matsumoto 3908510, Japan; Diabetes Center, Aizawa Hospital, Matsumoto 3908510, Japan; Department of Internal Medicine, Ina Central Hospital, Ina 3960033, Japan; Kidney Disease and Hemodialysis Center, Aizawa Hospital, Matsumoto 3908510, Japan; Kidney Disease and Hemodialysis Center, Aizawa Hospital, Matsumoto 3908510, Japan; Kidney Disease and Hemodialysis Center, Aizawa Hospital, Matsumoto 3908510, Japan; Health Center, Aizawa Hospital, Matsumoto 3908510, Japan; Health Center, Aizawa Hospital, Matsumoto 3908510, Japan; Diabetes Center, Aizawa Hospital, Matsumoto 3908510, Japan; Diabetes Center, Aizawa Hospital, Matsumoto 3908510, Japan

**Keywords:** plasma glucose, hemoglobin A1c, glycation, chronic kidney disease, general population

## Abstract

**Context:**

Chronic kidney disease (CKD) is a worldwide health problem. Recent literature has shown an association of hemoglobin glycation index (HGI) and CKD in patients with dysglycemia.

**Objective:**

The aim of this study was to reveal the impact of HGI as a predictor for incident CKD *in the general population*.

**Methods:**

CKD was defined as dipstick proteinuria or estimated glomerular rate (eGFR) < 60 mL/min/1.73 m^2^. Impact of HGI on incident CKD was assessed using the data from CKD-free health examinees (N = 23 467, 4.1% with diabetes) followed for a mean of 5.1 years: Cox proportional hazards model was employed with multivariate adjustment for age, systolic blood pressure, eGFR, fasting plasma glucose, body mass index, log[alanine aminotransferase], log[triglycerides], high-density lipoprotein cholesterol, platelet counts, smoking, and sex. Elevated level of HGI in subjects with CKD was ascertained after propensity score matching of another group of health examinees (N = 2580, 7.6% with diabetes).

**Results:**

In the former group, CKD developed in 2540 subjects and HGI was the second most robust predictor for CKD, following low eGFR. With adjustment for the 11 covariates, the hazard ratio of HGI (95% CI) for CKD was 1.293 (1.238 to 1.349) (*P <* .0001). The population attributable risk of HGI for CKD was 4.2%. In the latter group, among 708 subjects matched 1:1 for 9 covariates, HGI was significantly elevated in subjects with CKD (median [interquartile range] −0.208 [−0.504 to −0.156] vs −0.284 [−0.582 to 0.052], *P* = .03).

**Conclusion:**

HGI was a novel risk factor for CKD in the general population.

The increasing number of patients with chronic kidney disease (CKD) is a global concern (1-3). Treatment of established CKD is rather difficult and reversibility of the diseased kidney to the normal state is questionable. Therefore, prevention of CKD is of paramount importance; however, this is not easy to accomplish because a sizable number of patients with CKD develop it with elevated plasma glucose and/or mild hypertension, which are both asymptomatic at an early stage (4, 5). In an apparently healthy population, risk factor(s) for CKD are mostly unknown. Going over the historical data, reviews, and recent developments related to this topic, we observed that high hemoglobin glycation index (HGI) is significantly related to cardiovascular diseases and CKD (6-12) in patients with diabetes and prediabetes. HGI is a linear regression residual that is derived in 2 steps. First, a predicted glycated hemoglobin (HbA1c) value is generated by inserting the fasting plasma glucose (FPG) value into a regression equation describing the linear relationship between HbA1c and FPG in a given population. Predicted HbA1c is then subtracted from the individual's observed HbA1c (HGI = observed HbA1c − predicted HbA1c).

As an extension of such finding, we hypothesized that high HGI is a risk factor for incident CKD in the general population.

## Methods

### Study Population

We retrospectively analyzed the data from 2 independent groups of health examinees: one at Aizawa Hospital (Aizawa cohort) and the other one at Ina Central Hospital (Ina cohort).

There is a system called KENSHIN (13) in Japan. In this system, general citizens voluntarily come to a health center, usually to the same center, once a year, for which the employer is obliged to pay about half of the cost and the rest is paid by the participants. Thus, the participants in this study were relatively health-conscious people compared to those in the strictly speaking general population (14). Written informed consent was obtained from all participants, and the study was approved by the ethics committees of Aizawa Hospital and Ina Central Hospital.

### Definition of Chronic Kidney Disease and Diabetes Mellitus

In this study, CKD was defined as positive dipstick proteinuria and/or estimated glomerular filtration rate (eGFR) < 60 mL/min/1.73 m^2^ as in the previous studies (15-17). A single time positivity was regarded enough for the diagnosis of CKD (15-17). Diagnosis of diabetes was made if the FPG was higher than 126 mg/dL and/or the HbA1c higher than 6.5%.

### Outcome Measures

**Figure 1.**
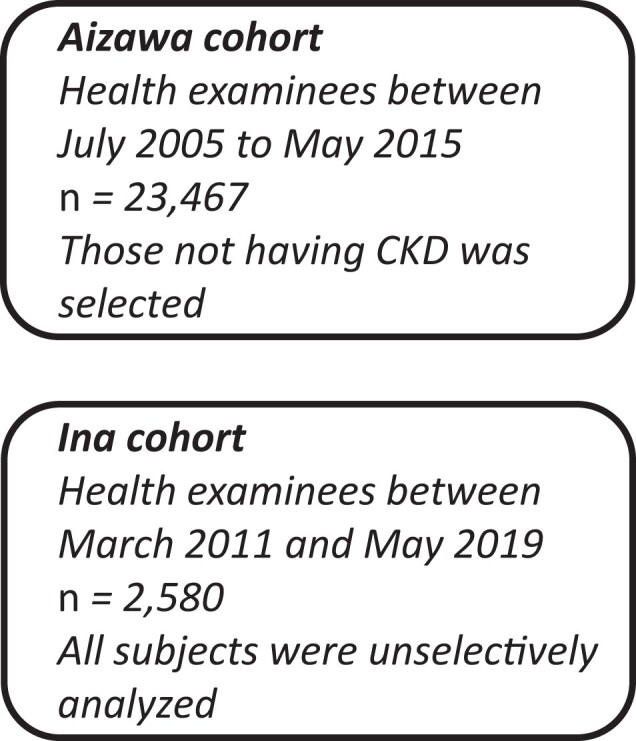
Flow of the study subjects.

In the Aizawa cohort (Fig. 1, upper half), development of CKD was taken as an endpoint and hazard ratios (HRs) of baseline HGI for incident CKD were calculated by the Cox proportional hazards model. As a secondary outcome, the impact of HGI on eGFR reduction rate was taken. The consistency of high HGI was ascertained by calculating HGI values of the participants in HGI quartile 4 over the initial 5 years of the study period. There was an interaction between HGI and basal eGFR, so that the Cox proportional hazards model was applied after stratification by basal eGFR as well. To confirm the results in the nondiabetic population, the analysis was also performed after exclusion of subjects with diabetes from the Aizawa cohort. In the Ina cohort (Fig. 1, lower half), the HGI level in subjects with and without CKD was compared. To this end, adjustment for age, systolic blood pressure, alanine aminotransferase (ALT), triglycerides (TG), high-density lipoprotein cholesterol (HDL-C), FPG, and body mass index (BMI) was performed by propensity score matching.

HGI is a linear regression residual that is derived in 2 steps. First, a predicted HbA1c is generated by inserting the FPG value into a regression equation describing the linear relationship between HbA1c and FPG in a given population. Predicted HbA1c is then subtracted from the individual's observed HbA1c; that is, HGI = observed HbA1c−predicted HbA1c.

The regression was, HbA1c (%) = 2.82 (95% CI, 2.79 to 2.86) + 0.028 (95% CI, 0.0279 to 0.0286) × FPG (mg/dL), (n = 23 467, *r*^2^ = 0.51, *P* < .0001) in the Aizawa cohort, and HbA1c = 2.84 (95% CI, 2.74 to 2.93) + 0.025 (95% CI, 0.024 to 0.026) × FPG (mg/dL), n = 2580, *r*^2^ = 0.54, *P* < .0001 in the Ina cohort.

The coefficients were not significantly different from those in the previous studies (6, 7). Participants were divided into 4 groups based on HGI quartile as needed in the multivariate analysis.

Estimated glomerular filtration rate (eGFR) was calculated by the equation developed for Japanese subjects (18, 19): eGFR (mL/min/1.73 m^2^) = 194 × serum creatinine (mg/dL)^−1.094^ × age (years)^−0.287^ (males), and eGFR (mL/min/1.73 m^2^) = [194 × serum creatinine (mg/dL)^−1.094^ ·age (years)^−0.287^] × 0.739 (females).

### Statistical Analysis

Analysis of the baseline characteristics shown in Table 1 was done by Wilcoxon signed rank sum test for numerical data, and comparisons of categorical data were made by the *x*^2^ test. Cox proportional hazards model was used for investigating the association between the CKD-free time of participants and HGI. The model included age, systolic blood pressure, eGFR, FPG, BMI, logALT, logTG, HDL-C, platelet count, smoking, and sex as covariates.

**Table 1.** Baseline characteristics and comparisons between progressors to CKD and non-progressors in the Aizawa cohort

Variable	All	Subgroups		*P values between progressors and non-progressors
		Progressors	Non-progressors	
*n* (male, %)	23 467 (14 020, 59.7%)	2540 (1653, 65.1%)	20 927 (12 567, 60.1%)	<.001
Age, years	49 (41 to 56)	54 (47 to 60)	48 (40 to 56)	<.001
BMI, kg/m^2^	22.7 (20.7 to 24.8)	23.4 (21.5 to 25.4)	22.6 (20.6 to 24.7)	<.001
SBP, mmHg	120 (110 to 131)	124 (114 to 136)	119 (109 to 130)	<.001
Smoking (current) %	8763 (37.3%)	878 (34.6%)	7885 (37.7%)	.0024
Triglycerides, mg/dL	88 (62 to 128)	99 (71 to 140)	87 (61 to 128)	<.001
ALT, IU/L	20 (15 to 28)	21 (16 to 28)	20 (15 to 28)	.0002
Platelet count, 10^4^/µL	22.4 (19.4 to 25.6)	21.5 (18.8 to 24.8)	22.5 (19.5 to 25.7)	<.001
FPG, mg/dL	94 (88 to 101)	95 (90 to 102)	94 (89 to 100)	<.001
HbA1c, % mmol/mol	5.5 (5.2 to 5.7) 36 (33 to 38)	5.6 (5.4 to 5.8) 38 (36 to 40)	5.5 (5.2 to 5.7) 37 (33 to 39)	<.001
HGI	−0.062 (−0.316 to 0.16)	−0.04 (−0.30 to 0.188)	−0.66 (−0.316 to 0.156)	.0018
eGFR, mL/min/1.73 m^2^	77.37 (69.91 to 84.93)	66.60 (63.12 to 70.84)	78.47 (71.59 to 85.91)	<.001

Data are median and IQR or *n* (%).

Abbreviations: ALT, alanine aminotransferase; BMI, body mass index; CKD, chronic kidney disease; eGFR, estimated glomerular filtration rate; HbA1c, glycated hemoglobin; FPG, fasting plasma glucose; HGI, hemoglobin glycation index; SBP, systolic blood pressure.

^*^ *P*, progressors vs non-progressors. Among progressors, 161/2540 (6%) were positive for dipstick proteinuria. Wilcoxon's signed rank test was used to compare numerical variables and x^2^ test was used to compare categorical variables.

Impact of nonlinearity of HGI on incident CKD was depicted by restricted cubic spline model. On the basis of the dose-response relationship between HGI and incident CKD, baseline HGI >0 (n = 10 112) was judged to be “exposure” and was used for calculation of percent attributable risk of HGI (20). The relationship between eGFR decline and HGI was assessed by a linear mixed model. In this analysis, the mean of 4.92 points/person was available for the calculation. Results are presented as regression coefficients and their standard errors. Effect of HGI was taken as a fixed effect on the slope of eGFR decline, which was calculated with participant ID as a random effect. The presence of interaction was checked by adding interaction term into the Cox model. At baseline, “HGI and eGFR” showed significant interaction (*P* = .03), but not “HGI and age,” “HGI and FPG,” and “HGI and gender.” Therefore, the HGI-CKD relationship was additionally analyzed after the binary stratification of the entire population by the baseline eGFR.

In the Ina cohort [Supplementary Table S1 (21)], propensity score matching was employed to adjust baseline differences between the subjects with and without CKD [Supplementary Table S2 (22)]. Logistic regression was used to generate the propensity scores. JMP Pro 16 version 2.0 was used for statistical analysis (JMP performs greedy nearest neighbor matching). *P* < .05 was considered statistically significant.

## Results

Baseline characteristics of the Aizawa cohort are shown in Table 1. Median age was 49 years, FPG was 94 mg/dL, HbA1c 5.5%, and eGFR 77.37 mL/min/1.73 m^2^. Incidence of CKD was 12 954 person-years. 1068 (4%) of the cohort had diabetes.

The hazard ratio (HR) of HbA1c for incident CKD was significantly lower than that of HGI both in univariate and bivariate analysis (Table 2). Since the HGI was a far more robust risk factor than HbA1c, the analysis was focused on HGI in the rest of the study. The HR per 1 unit of HGI was 1.82 (95% CI, 1.69 to 1.95) (Table 2).

**Table 2.** Hazard ratio and 95% CI of HGI and HbA1c to incident CKD

	HR	95% CI
Univariate analysis		
HGI	1.826	1.696 to 1.956
HbA1c	1.397	1.339 to 1.456
Bivariate analysis		
HGI	1.612	1.403 to 1.852
HbA1c	1.097	1.005 to 1.196

Data were obtained from the Aizawa cohort, in which IQR of HGI and HbA1c was almost the same as 0.48 and 0.50 (Table 1) so that scaling was omitted.

Abbreviations: CKD, chronic kidney disease; eGFR, estimated glomerular filtration rate; HbA1c, glycated hemoglobin; HGI, hemoglobin glycation index.

The relationship between HGI and CKD was highly significant even after adjustment for the covariates including FPG (Table 3). The risk was progressively greater with increasing HGI so that subjects at quartile 4 of HGI at baseline were at 1.7 times higher risk compared to those in quartile 1 (Table 3). Other than high HGI, elevated blood pressure, low HDL-C and high TG were also significant predictors of CKD (Table 3).

**Table 3.** Relationship between potential risk factors and incident chronic kidney disease

Model 1. Entire population (n = 23 467)			Model 2. Nondiabetic population (n = 22 412)		
Risk factor	Relationship between CKD and risk factor		Risk factor	Relationship between CKD and risk factor	
	Adjusted HR (95% CI) Scaled to 1 IQR	*P* value		Adjusted HR (95% CI) Scaled to 1 IQR	*P* value
HGI	1.284 (1.229 to 1.341)	<.001	HGI	1.332 (1.256 to 1.413)	<.001
HGI quartile (n, range)			HGI quartile (n, range)		
Q1 (5876, −3.580 to −0.316)	1.0 (Ref)		Q1 (5766, −2.190 to 0.274)	1.0 (Ref)	
Q2 (5855, −0.314 to −0.064)	1.319 (1.177 to 1.478)		Q2 (5778, −0.026 to −0.224)	1.296 (1.154 to 1.456)	
Q3 (5884, −0.062 to 0.160)	1.459 (1.301 to 1.635)		Q3 (5765, −0.226 to 0.448)	1.534 (1.366 to 1.723)	
Q4 (5852, 0.162 to 4.888)	1.774 (1.582 to 1.988)		Q4 (5083, 0.450 to 1.474)	1.811 (1.609 to 2.038)	
Age, years	1.178 (1.095 to 1.271)	<.0001	Age, years	1.141 (1.057 to 1.232)	<.0001
SBP, mmHg	1.138 (1.074 to 1.206)	<.0001	SBP, mmHg	1.204 (1.097 to 1.317)	<.0001
eGFR, mL/min/1.73 m^2^	0.115 (0.104 to 0.127)	<.0001	eGFR, mL/min/1.73 m^2^	0.103 (0.095 to 0.11)	<.0001
FPG, mg/dL	1.042 (0.990 to 1.052)	0.190	FPG, mg/dL	1.008 (0.958 to 1.061)	.130
BMI, kg/m^2^	1.044 (0.979 to 1.113)	0.191	BMI, kg/m^2^	1.050 (0.984 to 1.126)	.140
logALT, IU/L	0.946 (0.891 to 1.003)	0.364	logALT, IU/L	0.819 (0.599 to 1.122)	.213
logTG	1.015 (0.943 to 1.092)	<0.001	logTG	1.015 (0.943 to 1.092)	.699
HDL-cholesterol	0.905 (0.835 to 0.960)	<.001	HDL-cholesterol	0.866 (0.819 to 0.931)	<.001
Platelets, 10^9^/L	1.047 (0.976 to 1.123)	.203	Platelets, 109/L	0.884 (0.837 to 0.934)	<.001
Smoking (+) (−)	1.191 (1.089 to 1.303) (−) (Ref)	<.001	Smoking (+) (−) (Ref)	1.115 (1.009 to 1.233) 1.0 (Ref)	.006
Sex (Female) (Male)	0.893 (0.846 to 0.942) 1.0 (Ref)	0.017	Sex (Female) 1.0 (Male)	1.164 (1.078 to 1.257) 1.0 (Ref)	.0033

HR was adjusted for the listed covariates, and it was scaled to the interquartile range (IQR) (computed by subtracting the 25th percentile from the 75th percentile and defined as the unit for HR). Such scaling was not applied for the categorical variables: HGI quartile (shown in the rectangle drawn on the top), smoking, and sex. Note that *increased* eGFR is a protective factor so that risk from *decreased* eGFR can be obtained by calculating reciprocal of the listed HR.

Abbreviations: ALT, alanine aminotransferase; BMI, body mass index; CKD, chronic kidney disease; eGFR, estimated glomerular filtration rate; HbA1c, glycated hemoglobin; HR, hazard ratio; FPG, fasting plasma glucose; HDL, high-density lipoprotein; HGI, hemoglobin glycation index; SBP, systolic blood pressure; TG, triglycerides.

**Figure 2.**
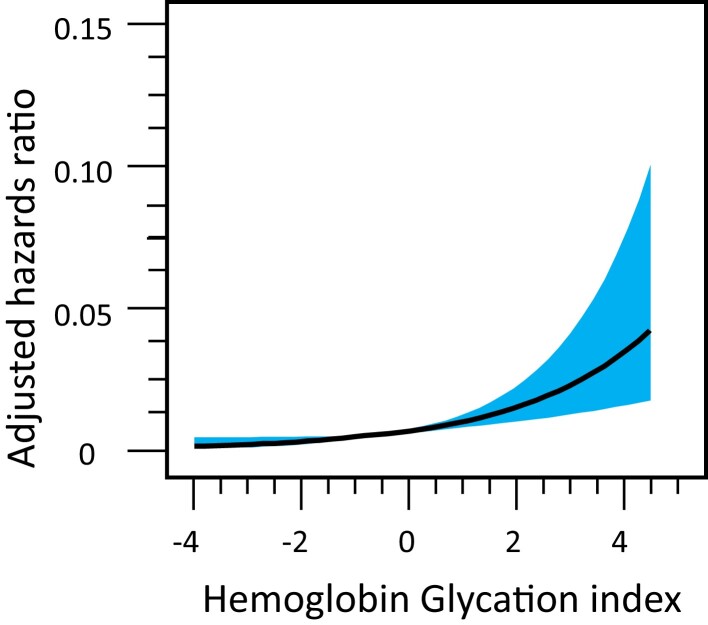
Dose-effect of HGI on the actual incidence of CKD in the Aizawa cohort. The vertical axis indicates actual incident CKD/5.1 years and the horizontal axis is baseline HGI, smoothed by the cubic spline method. The black line represents the mean; the shaded area surrounding it area, 95% CI.

To obtain in-depth understanding on the nature of the relationship between dose (exposure) and effect (incident CKD), we used a cubic spline model with 5 knots (5th, 27.5th, 50th, 72.5th, and 95th percentiles) (Fig. 2) (*P* overall association <.0001; *P* nonlinearity > .99). On the basis of this dose-response relationship, those with baseline HGI greater than 0 (n = 101 122) were regarded to be “exposed to the risk (in this study higher HGI).” Then, the population attributable risk (20) of HGI for incident CKD was calculated to be 4.2%. HGI was slightly but significantly more robust than aging as a risk factor for CKD (Table 3, Model 1). Such strong impact of HGI on incident CKD was evident even when subjects with diabetes were excluded (Table 3, Model 2). Namely, multiple adjusted HR for CKD scaled for interquartile range (IQR) was slightly but significantly greater than that of aging (Table 3, Model 2).

HGI showed an inverse correlation with eGFR: regression coefficients and their standard error were −0.39 ± 0.08, which means that the multi-adjusted eGFR reduction/year was 0.39 mL/min/0.73 mL/m^2^ greater per 1.0 increase in baseline HGI. The consistency of HGI was checked by the retention rate of subjects belonged to quartile 4 of HGI: it was 65%, 63%, 50%, and 47% at year 2, 3, 4, and 5, respectively. These data indicated that elevated HGI is not a chance, but rather a persistent, phenomenon.

**Figure 3.**
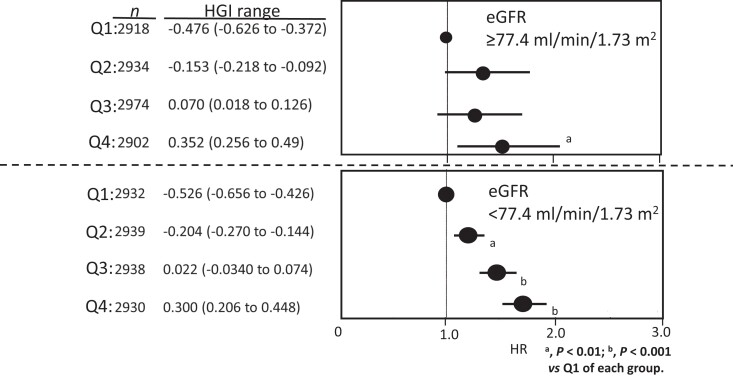
Multi-adjusted HR of HGI quartile for incident CKD in the subjects stratified by baseline eGFR in the Aizawa cohort. The entire Aizawa cohort was binary split into upper (n = 11 639) and lower (n = 11 828) halves of eGFR. The upper and the lower panels show the results from those with higher and lower halves of eGFR, respectively.

The HGI-CKD relationship was additionally analyzed after stratification by baseline eGFR. This was performed because there is a significant interaction between HGI and eGFR. HGI was clearly a dose-related risk factor for incident CKD in subjects with lower but not higher eGFR (Fig. 3). In the Ina cohort, the HGI level of subjects with and without HGI was compared after propensity score match adjustment for the covariates. It was significantly greater in subjects with CKD than those without it: −0.208 (−0.535 to −0.156) vs −0.284 (−0.582 to 0.052), *P* = .039 (Supplementary Table S2) (22).

## Discussion

A seminal finding in this study was that HGI was an independent, robust, and reliable risk factor for incident CKD. The finding is novel in 3 aspects. First, HGI predicted CKD among the apparently healthy, general population. Second, HGI was the most robust risk factor except for lowered GFR. Third, and most importantly, the population attributable risk of HGI for CKD was about 4% for incident CKD. Up to now, we have had only limited tools to predict future CKD for individuals who do not suffer from conventional risk factors, such as diabetes and hypertension (4, 5, 15-17). From this point of view, the 4.2% is not a small figure, because the denominator is the apparently healthy general population. This study was a retrospective observational analysis. However, reverse causality is most unlikely because reduced kidney function causes lowered, not elevated, HbA1c values (23). Our results imply that high HGI may be an important measurement in the general population for prediction of incident CKD. Of note, the incidence of CKD in the Aizawa cohort was approximately 2% per year which is smaller than in the previous studies: (3.5% to 20% per year) (16, 17). The favorable data in our cohort might be due to lower HGI of the Aizawa cohort than the previous cohorts (16, 17), but HGI was not obtained in the previous studies (16, 17). In our study, age was younger, blood pressure was lower, and the prevalence of diabetes was lower. It was most unlikely that a substantial number of subjects in the Aizawa cohort had Stage 3 or 4 CKD already at the baseline because the incidence of those with microalbuminuria; that is, subjects with Stage 3 or higher (24, 25) is reported to be 10%. In the Aizawa cohort, hypertension, low HDL-C and high TG were weak, but significant predictors of CKD. This suggested that so-called metabolic syndrome may also have been playing a role in the genesis of the early phase renal impairment, even in this cohort with metabolically favorable features (26). Lack of data on abdominal obesity hindered further exploration of this.

Our data on HGI likely opens a new horizon as the bridging between metabolism, diabetes, and nephrology. From a viewpoint of diabetologist, the “normal range” of HbA1c needs to be critically reconsidered in relation to the current data: even in the range of FPG < 100 mg/dL, elevated HbA1c relative to glucose was a definite predictor of incident CKD. For nephrologists, this observation in subjects without diabetes but with elevated HGI, should be regarded as an important, previously unrecognized, source of CKD. Preferential glycation of a certain renal key component *in the face of normoglycemia* is a possible mechanism for the link between high HGI and incident CKD. In this regard, a recent report on telomere attrition associated with high HGI is an attractive candidate (27). Although HbA1c is generally regarded as a reliable indicator of chronic glycemia, a certain fraction of people consistently show higher HbA1c values than others, in the presence of comparable level of glycemia (7, current study]. We consider that future studies on the CKD mechanism would clarify the underlying pathophysiological significance of glycation under normoglycemia. Currently, abnormal (excess) glycation is discussed solely as a disease process taking place under hyperglycemia (28). HGI was highly correlated with HbA1c levels, which might be a concern. Namely, one may suspect that HGI just worked as an alternative to (or marker for) HbA1c levels. If this were the case, all one has to do is simply use HbA1c levels as they are. Yet, we believe this was not the case. In Table 3, the results of head-to-head comparison of HGI and HbA1c are shown. The data showed that HGI was a significantly stronger predictor than HbA1c for incident CKD.

### Strength of the Study

This was a systematic analysis in a large number of apparently healthy individuals, regarding a novel, simple, and clinically usable index. The concept was straightforward and well supported by the data, therefore possesses a strong clinical impact.

### Limitation of the Study

This is a retrospective study so a certain degree of unintentional selection bias cannot be ruled out, and we could not perform a direct measurement of GFR. Pathological evaluation of the kidney also was not carried out. The external validation is lacking and so is biochemical approach to the mechanistic link between HGI and CKD. We judged those with FPG ≥ 126 mg/dL and/or HbA1c higher than 6.5% as having diabetes and excluded these people from the subanalysis. Therefore, patients already treated, and having excellent glycemic control (FPG < 126 mg/dL and HbA1c lower than 6.5%), were included in the subanalysis. This is a study exclusively on Japanese subjects, so that applicability of the data in other ethnic groups remains to be examined.

## Conclusion

HGI was a robust predictor of incident CKD in an apparently healthy, nondiabetic cohort. There was an interaction between HGI and eGFR and the binary stratification of the study subjects by basal eGFR revealed that the prediction was mostly taking place among those with lower half of eGFR (eGFR <77.4 mL/min/1.73 m^2^). The population attributable risk of HGI was 4.2%. It is most likely that high HGI is a novel risk factor for CKD.

## Data Availability

Original data generated and analyzed during this study are included in this published article.

## Abbreviations

ALT: alanine aminotransferase
BMI: body mass index
CKD: chronic kidney disease
eGFR: estimated glomerular filtration rate
HbA1c: glycated hemoglobin
HDL-C: high-density lipoprotein cholesterol
HR: hazard ratio
FPG: fasting plasma glucose
HGI: hemoglobin glycation index
TG: triglycerides
