# Correction to “Hemoglobin Glycation Index: A Novel Risk Factor for Incident Chronic Kidney Disease in an Apparently Healthy Population”

**DOI:** 10.1210/clinem/dgae066

**Published:** 2024-02-08

In the above-named article by Nakasone Y, Miyakoshi T, Sakuma T, Toda S, Yamada Y, Oguchi T, Hirabayashi K, Koike H, Yamashita K, and Aizawa T (*J Clin Endo Metab*. 2023; doi: 10.1210/clinem/dgad638), there were errors in the text as published:

In Table 1, the entries in the row “eGFR, mL/min/1.73 m^2^” and columns “Progressors” and “Non-progressors” were switched. The entry in the “Progressors” column should read “66.60 (63.12 to 70.84)” and the entry in the “Non-progressors” column should read “78.47 (71.59 to 85.91).”

In the “Results” section, the following text appeared: “Namely, multiple adjusted HR for HGI scaled for interquartile range (IQR) was slightly but significantly greater than that of aging (Table 3, Model 2).” The sentence should read: “Namely, multiple adjusted HR for CKD scaled for interquartile range (IQR) was slightly but significantly greater than that of aging (Table 3, Model 2).”

The article has been corrected online.

doi: 10.1210/clinem/dgad638

